# Determination and benchmarking of ^27^Al(d,α) and ^27^Al(d,p) reaction cross sections for energies and angles relevant to NRA

**DOI:** 10.1038/s41598-021-97372-7

**Published:** 2021-09-10

**Authors:** M. Salimi, O. Kakuee, S. F. Masoudi, H. Rafi-kheiri, E. Briand, J.-J. Ganem, I. Vickridge

**Affiliations:** 1 Department of Physics, K.N. Toosi University of Technology, P.O. Box 15875-4416, Tehran, Iran; 2 Sorbonne Université, CNRS, Institut des NanoSciences de Paris, INSP, SAFIR, 75005 Paris, France; 3 Physics and Accelerators Research School, NSTRI, P.O. Box 14395-836, Tehran, Iran

**Keywords:** Nuclear physics, Experimental nuclear physics

## Abstract

The cross-sections of deuteron-induced nuclear reactions suitable for ion beam analysis, measured in different laboratories, are often significantly different. In the present work, differential cross-sections of ^27^Al(d,p) and ^27^Al(d,α) reactions were measured, and the cross sections benchmarked with thick target spectra obtained from pure aluminium for the first time in two independent laboratories. The ^27^Al(d,p) and (d,α) differential cross-sections were measured between 1.4 and 2 MeV at scattering angles of 165°, 150°, and 135° in the VDGT laboratory in Tehran (Iran), and the same measurements for detector angle of 150° were repeated from scratch, including target making, with independent equipment on the SAFIR platform at INSP in Paris (France). The results of these two measurements at 150° are in good agreement, and for the first time a fitted function is proposed to describe the Al-cross sections for which no suitable theoretical expression exists. The obtained differential cross-sections were validated through benchmarking, by fitting with SIMNRA deuteron-induced particle spectra obtained from a high purity bulk Al target at both labs for deuteron incident energies between 1.6 and 2 MeV. The thick target spectra are well-reproduced. The evaluated and benchmarked cross sections have been uploaded to the ion beam analysis nuclear data library database (www-nds.iaea.org/ibandl/).

## Introduction

Ion beam analysis (IBA) has been widely used to analyze quantitatively and with high sensitivity the composition and elemental depth profiles in the surface regions of solids. For light element analysis, suitable nuclear reactions can be found and in particular, deuteron-induced reactions, (d,p) or (d,α), often have high Q-values and appreciable cross-sections. In many cases of Nuclear Reaction Analysis (NRA) of thin films, isolated reaction particle peaks may be obtained with judicious choice of scattering angle, incident beam energy, and filtering foils in front of the charged particle detector. However, in general, NRA generates complex spectra with overlapping peaks, especially from thick samples.

Knowledge of the cross-sections for the cases of isolated peaks is already useful for designing experiments to determine elemental contents of thin films. Many such cross-sections, for example ^16^O(d,p_0_)^17^O, ^16^O(d,p_1_)^17^O, ^12^C(d,p_0_), ^14^N(d,α_0_), have been carefully measured at energy and angular ranges of analytical interest^1–4^. It is sometimes possible to analyse several light elements simultaneously by NRA. Knowledge of the cross-sections of reactions that are not necessarily of primary interest for thin film analysis, is then often needed for cases in which targets contain elements giving rise to reactions that produce particle groups that interfere with the primary analytical peak, and even more so in thick target NRA where the broadening of the particle spectra from the non-zero target thickness leads to a much greater probability of elemental interferences^5–7^.

The need for accurate cross sections, even when not of primary interest for a specific analytical problem, or in energy ranges that are not directly analytically useful, has also recently become more acute, with the introduction of the concept of Total IBA^8–10^ in which all information from IBA spectra is exploited, and the growing use of Artificial Intelligence and machine learning approaches to optimise information extraction from all parts of IBA spectra^11^. So far, Artificial Neural Networks (ANN) have been applied for the case of Rutherford Backscattering Spectrometry, where the cross sections are known analytically, however reliable extension of these advanced data treatment techniques to NRA requires the best possible nuclear reaction cross sections. Furthermore, accurate experimental nuclear reaction cross-sections are required to provide proper parameters for appropriate approximations and expressions of theoretical nuclear reaction mechanism models.

Because oxygen is the most abundant element in the earth’s crust and because of the universal importance of oxides in earth sciences and materials science, accurate cross-sections for ^16^O and ^18^O nuclear reactions have been determined^1,12^. The second most abundant element is silicon, and although it is an intermediate-mass element from the point of view of IBA, it also has nuclear reactions of analytical interest that have been determined^2,13^.

Aluminium is the third most abundant element that is widely used in industry for its mechanical and electrical properties, decorative applications, and its resistance to environmental aggression especially after suitable electrochemical passivation. Aluminium is also widely present in the alumina-silicate rocks that constitute so much of the earth’s upper crust. In many thin film systems, such as III-Nitrogen semiconductors developed amongst other things for UV-C light emitting diodes destined to replace UV mercury lamps in mass sterilisation applications for COVID mitigation, and thin conformal alumina films or nanolaminates grown by Atomic Layer Deposition where aluminium is an essential component, absolute determination of the Al content is paramount for developing improved materials. There is a wealth of deuteron-induced charged particle reactions on ^27^Al with high Q-values that may be exploited in NRA for analysis of aluminum or encountered as interferences in NRA of aluminium-containing materials^14–24^.

There is a further motivation for good knowledge of Al cross sections: even if analysis of Al is not the main objective of a measurement, reaction products from Al may interfere with the signals from other reactions of interest and to be able to fully fit a complex NRA spectrum, all of the cross sections involved need to be known. Detailed knowledge of the cross-sections of these reactions is thus a significant interest in the field of NRA. The compound nucleus ^29^Si contains too many close levels to be able to be treated satisfactorily within R-Matrix theory, and too few to be able to be handled satisfactorily with a statistical approach. At present, the best we can obtain is well measured experimental cross-section^14–20^.

The first measurements of ^27^Al(d,α) and ^27^Al(d,p) cross-sections were made by Gadioli^25^ at 150° as part of a study of the fluctuation mechanism occurring in the differential excitation functions. These cross-section curves have since been measured for deuteron energies less than 3 MeV at 90°, 135°, 150°,165°, and173°^14,17–20,25–27^, however significant gaps and discrepancies remain.

In this work, we have determined the cross-sections for the ^27^Al (d,p_0+1,2+3,4,5+6,9,10,11,12_) and ^27^Al(d, α_0,1,2,3,4_) reactions at an energy below 2 MeV and at 150° scattering angle in two completely independent measurements in two different laboratories: the Van De Graaff Lab in Tehran (VDGT) and the SAFIR platform of the Institut des NanoSciences de Paris (INSP). The ^27^Al(d,p) and (d,α) cross-sections were also determined at 135° and 165° at VDGT. From our measured data and a critical evaluation of previous measurements, we propose a set of recommended cross-sections for NRA and demonstrate their validity through benchmarking experiments in a thick pure aluminum target in both laboratories.

## Experimental setup and procedure

The experimental setups at VDGT and INSP are significantly different. We present here the main experimental features of each laboratory.

### Experimental setup and procedure at VDGT

#### Chamber and data acquisition

At VDGT, cross sections and benchmarking spectra were measured in the 30° left beamline of the 3 MV Van de Graaff electrostatic accelerator, equipped with a chamber developed for accurate and reliable RBS/NRA measurements^28^. A beam of 20–40 nA was directed into a beam spot of 1.5 × 1.5 mm^2^. The energy resolution is estimated to be about 1 keV. Under these conditions, deadtime was less than 10% and pileup was minimized.

The pressure of the chamber was about 2 × 10^–6^ mbar during the measurements. The detection system for all of the measurements consisted of three 25 mm^2^ × 300 µm thick surface barrier detectors installed at 135°, 150°, and 165° degrees from the incident beam direction. The angular spread of each detector was less than 3°, with solid angles Ω of 1–2 msr. Integrated beam charge Q of 10 μC was usually sufficient to obtain adequate statistics.

#### Calibration energy of the accelerator

We determined beam energy from the field strength of the analyzing magnet, measured with an NMR fluxmeter^29^. The energy calibration of the accelerator was determined from the reaction threshold energy at 1880.44 ± 0.02 keV in the ^7^Li(p,n)^7^Be reaction. The target was a pressed LiF pellet with a 10 µg/cm^2^ silver coating for charge evacuation and neutrons were detected with a BF3 detector.

#### Target preparation

The thin Al target must be of appropriate thickness, stable both in atmosphere and under the beam in vacuum, and should also be amorphous to avoid unwanted channeling effects^30^. To achieve these requirements, physical vapor deposition (PVD) was chosen from amongst the different methods for thin Al target preparation^31^. Pure Al was evaporated onto a microscope slide which had been prepared by dipping into a mixture of water and detergent under ultrasonic agitation. The PVD system was a model VE-770 with a base pressure of about 10^–6^ mbar, equipped with a coiled tungsten filament. The obtained film was floated onto water and then fished over an 8 mm diameter hole in a thin metal sheet. Finally, 10 ± 0.5 nm of Ag was deposited onto the thin self-supporting vacuum evaporated Al film as an internal reference^28,32^.

#### Characterization of the thin target

The thin target thickness is determined by RBS^33^ with an uncertainty of less than 5%, due to uncertainties in Q × Ω, in possible deviations of the ^27^Al(α,α)^27^Al cross section from the assumed Rutherford cross section and in fitting the simulated spectrum to the measured spectrum. The thickness of target was only used for determination of deuteron beam energy loss through the target. The thickness and stoichiometry of the Al/Au target were measured at the three detection angles of 135°, 150°, and 165° by alpha particle beams of 1.8 MeV. This measurement was simulated with SIMNRA 7.03 by using the Chu and Yang straggling model and Ziegler/Biersack stopping power^34^. Typical simulated and measured spectra are shown in Fig. 1.

**﻿Figure 1:**
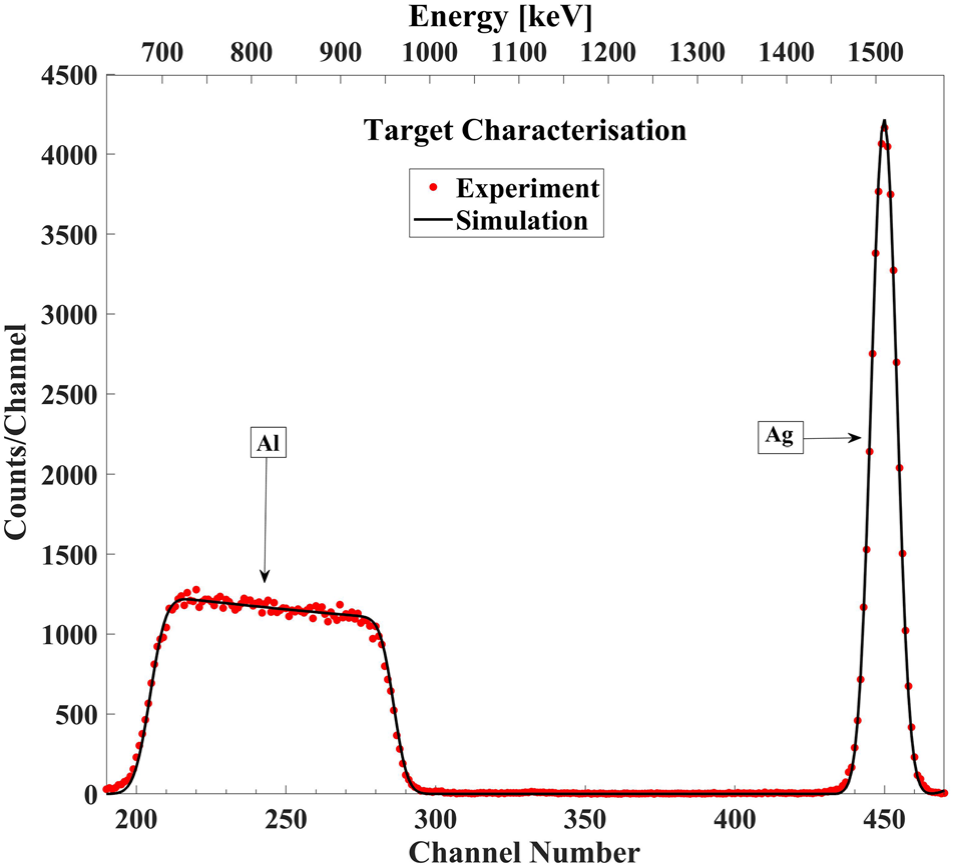
Typical simulated and measured RBS spectra from a thin Al/Ag target obtained at scattering angle 150° and E_α_ = 1.8 MeV.

In target characterization with the alpha beam, the areal density ratio $\frac{{N_{{{\varvec{Ag}}}} }}{{{\varvec{N}}_{{{\varvec{Al}}}} }}$ was calculated by equation^1^:

1 $$\frac{{N_{Ag} }}{{N_{Al} }} = \frac{{Y_{Ag} }}{{Y_{Al} }} \times \frac{{\left( {\frac{d\sigma }{{d\Omega }}} \right)_{{\theta ,EBS,E = E_{Al,\alpha } }}^{Al} }}{{\left( {\frac{d\sigma }{{d\Omega }}} \right)_{{\theta ,Ruth,E = E_{Ag,\alpha } }}^{Ag} }}$$

where *N* corresponds to the areal density of the target and *Y* to the experimental yield (net area under the peak). Here, *N*_*A*_ rather than [A] is used to represent the areal density of species A, in order to simplify the expressions. The average of these ratios from different detection angles was estimated by assuming the Rutherford cross section for reaction of alpha particles with *Ag* and *Al*, and applying a correction for the energy loss in the thin target. $\left( {\frac{d\sigma }{{d\Omega }}} \right)_{{\theta ,Ruth, E = E_{Ag,\alpha } }}^{Ag}$ is defined as the Rutherford cross section at $E_{Ag,\alpha } = E_{\alpha } - \frac{{ \left( {\Delta E } \right)_{\theta , E\alpha }^{{{\text{Ag}}}} }}{2}$ for *E*_α_ = 1.8 MeV. The alpha particle elastic scattering cross section $\left( {\frac{d\sigma }{{d\Omega }}} \right)_{{\theta ,EBS, E = E_{Al} ,\alpha }}^{Al}$ can be considered to be Rutherford for the energy $E_{Al,\alpha } = \widetilde{{ E_{Al} }} - \frac{{ \left( {\Delta E } \right)_{{{\theta }, E = \widetilde{{E_{Al} }}}}^{{{\text{Al}}}} }}{2}$; $\widetilde{{ E_{Al} }} = E_{\alpha } - \left( {\Delta E } \right)_{{{\uptheta }, E = E\alpha { }}}^{{{\text{Ag}}}}$, where *E*_α_, $\left( {\Delta E } \right)_{{{\theta }, E = \widetilde{{E_{Al} }}}}^{{{\text{Al}}}}$ and $\left( {\Delta E } \right)_{{{\uptheta }, E\alpha { }}}^{{{\text{Ag}}}}$ are the incident alpha energy, energy loss in the Al layer for $E = \widetilde{{ E_{Al} }}$ and energy loss in *Ag* layer, respectively.

Using this ratio eliminates uncertainty due to solid angle, dead time, and charge measurement for the differential nuclear cross section measurements.

### Experimental work in INSP lab

At INSP Lab in Paris, the 30° right beamline of the 2.5 MV Van de Graaff electrostatic accelerator of SAFIR (Système d’Analyse par Faisceaux d’Ions Rapides) platform was employed for our measurement.

The accelerator energy, read from the Generating Voltmeter signal, was calibrated by using the narrow nuclear resonances of the ^27^Al (p, γ) ^28^Si reaction at 991.88 keV, the ^13^C(p,γ)^14^N reaction at 1747.6 keV and the ^15^N(p, αγ)^12^C reaction at 426.1 keV. In each case, the gamma rays were detected with a BGO scintillation detector at 0°^35,36^. Because of the very high energy resolution of the beam, less than 250 eV full width half maximum energy spread over the range reported here, excitation curves were fitted with SPACES^37^ so as to compensate for small distortions of the excitation curve due to surface contaminants and oxidation. The treatment of the GVM signal has been implemented in Labview rather than in analogue electronic circuits, giving an energy calibration that is highly linear and practically independent of temperature.

**Figure 2:**
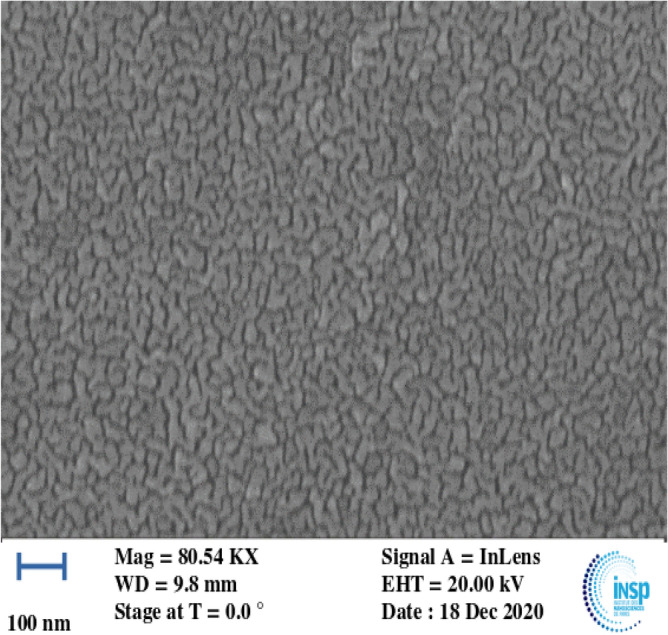
SEM images of the surface of from Au/Al target.

A similar method to that used at VDGT was employed at INSP for preparation of the thin self-supporting targets. An EDWARDS FL 400 and VINCI technologies PVD 4E were used for Al and Au evaporation respectively. The preparation—floating and fishing—and characterization of the target were the same as at VDGT Lab in Tehran. The heavy element used as internal standard need only provide a sufficiently well separated peak of adequate intensity, and here we have used Au rather than Ag as the internal standard for the INSP targets since it was more conveniently available. Subsequently the areal density ratio was taken into account as $\frac{{N_{{{\varvec{Au}}}} }}{{{\varvec{N}}_{{{\varvec{Al}}}} }}$ in the differential cross section measurement, and of course the appropriate energy loss was calculated for the gold layer. Also, absolute values of the areal densities *N*_*Al*_ and *N*_*Au*_ were determined by comparing with the standard Bi-implanted Si reference with an uncertainty of 2%–3% in the same RBS measurement. Scanning electron microscopy (SEM) images of the surface of from Au/Al target, shown in Fig. 2, confirmed the uniformity of the surface structure of samples at the nanoscale.

## Data analysis and results

### The differential nuclear cross section measurement value at VDGT Lab in Tehran

Among the nuclear reactions that can be used to characterize Al, ^27^Al(d,p_0+1,2+3,4,5+6,9,10,11,12_)^28^Al and ^27^Al(d,α_0,1,2,3,4_)^25^Mg were selected for measurements of the deuteron induced reaction cross sections since these particle groups can be identified and their intensities estimated under reasonable experimental conditions. The cross sections were measured with 10 keV steps, for energies ranging from 1.3 to 2 MeV.

**Figure 3:**
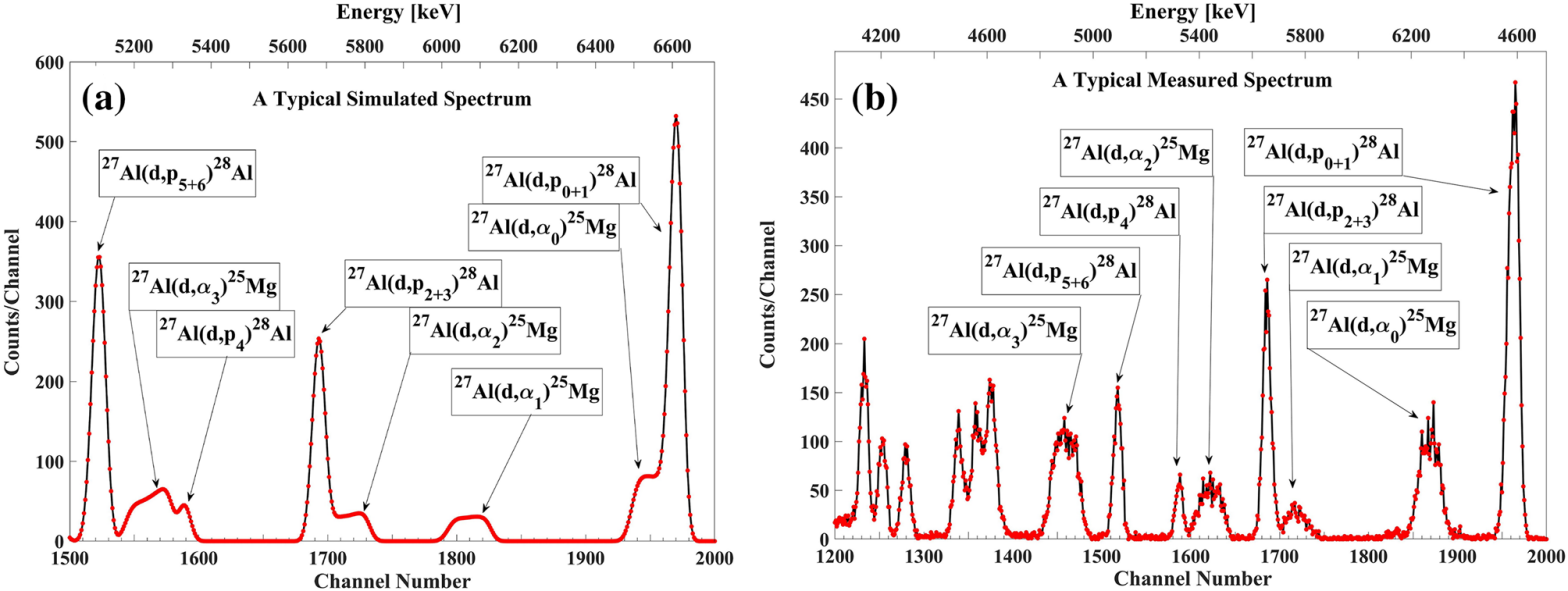
(**a**) The simulated spectrum without mylar and (**b**) the typical measured spectrum in experimental work with 4 µm mylar in front of the particle detector at the detection angle of 150° and for E_d,lab_ = 1.8 MeV at VDGT Lab in Tehran. The interferences between the high energy alpha and proton groups are clearly resolved.

In order to discriminate between high Q-value (d,p) and (d,α) groups in a single measurement setup, we placed a 4 µm thick mylar filter in front of the detector. The judicious choice of 4 μm mylar thickness, to minimize interferences between proton and alpha particle groups in the thin target spectra, was initially found through SIMNRA simulations. The discrimination is illustrated in Fig. 3, comparing a real measured spectrum with 4 μm mylar, with a simulated spectrum without filter, clearly confirming experimentally that interference between the particles from ^27^Al(d,p_0+1_)^28^Al and ^27^Al(d,α_0_)^25^Mg, and from ^27^Al(d,p_4_)^28^Al and ^27^Al(d,α_3_)^25^Mg is eliminated.

The differential cross section values $\left( {\frac{{{\text{d}}\sigma }}{d\Omega }} \right)_{\theta ,E(Al)}$ at the energy *E*_*d*_ and detection angle *θ* were obtained from Eq. (2):

2 $$\left( {\frac{{{\text{d}}\sigma }}{d\Omega }} \right)_{\theta ,E(Al)} = \left( {\frac{d\sigma }{{d\Omega }}} \right)_{{\left. {\theta ,EBS,E_{d} (Ag} \right)}}^{Ag} \times \frac{{Y_{Al} }}{{Y_{Ag} }} \times \frac{{N_{Ag} }}{{N_{Al} }} \times \frac{{(Q \times \Omega )^{\theta ,Ag} }}{{(Q \times \Omega )^{\theta ,Al} }}$$

where *E*_*d*_, *Y*_*Al* or Ag_, *θ* and $\left( {\Delta E } \right)_{{{\theta },d}}^{{{\text{Ag}}}}$ represent incident deuteron beam energy, the experimental yield of *Al* or *Ag* (net area under the peak), scattering angle and energy loss in *Ag* layer, respectively. $\left( {\frac{d\sigma }{{d\Omega }}} \right)_{{\left. {\theta ,EBS,E_{d} (Ag} \right)}}^{Ag}$ is the differential cross section of the ^*nat*^*Ag*(*d*,*d*_*0*_) reaction at $\left. {E_{d} (Ag} \right) = E_{d,\theta } - \frac{{ \left( {\Delta E } \right)_{\theta ,d}^{Ag} }}{2}$, which is Rutherford below 2 MeV. $\frac{{(Q \times \Omega )^{\theta ,Ag} }}{{(Q \times \Omega )^{\theta ,Al} }}$ is equal to one since the charge (*Q*) and solid angle (*Ω*) for *Al* and *Ag* are identical at each scattering angle, eliminating the uncertainty due to *Q* and *Ω* measurement. Also, $\frac{{N_{Ag} }}{{N_{Al} }}$ was measured by RBS as expl*a*ined above.

**Figure 4:**
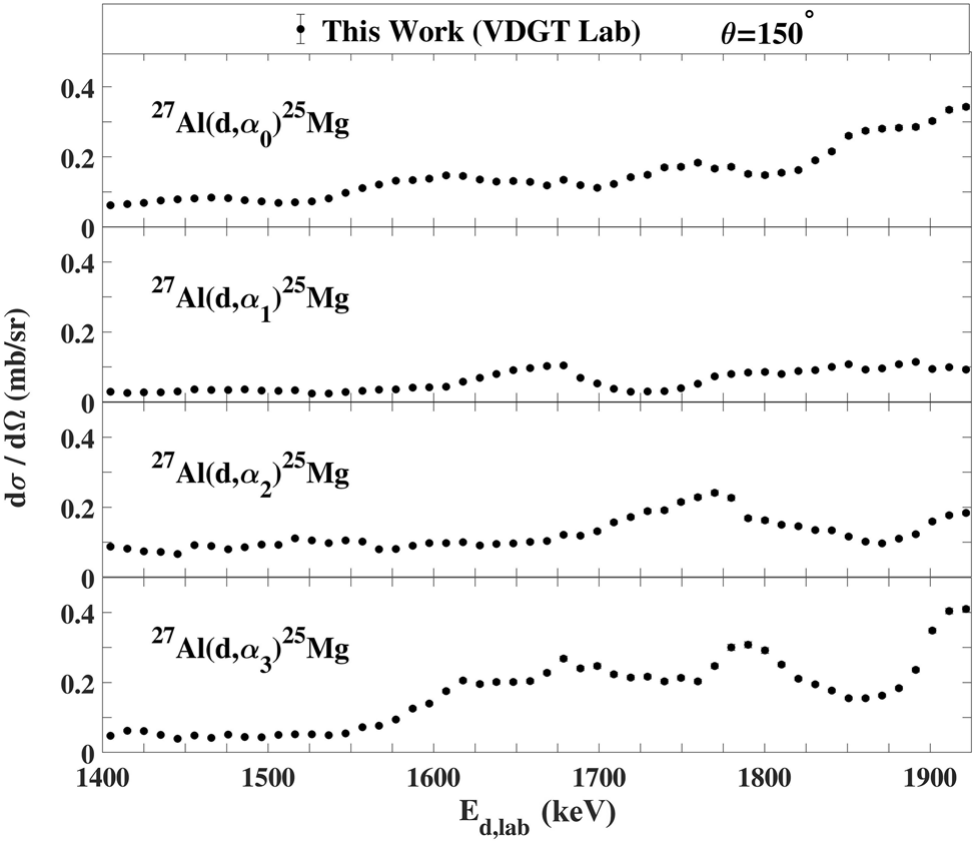
The excitation functions at scattering angle 150° at VDGT Lab in Tehran for the ^27^Al(d,α)^25^Mg reactions. The estimated uncertainties from counting statistics are smaller than the plotted symbols.

**Figure 5:**
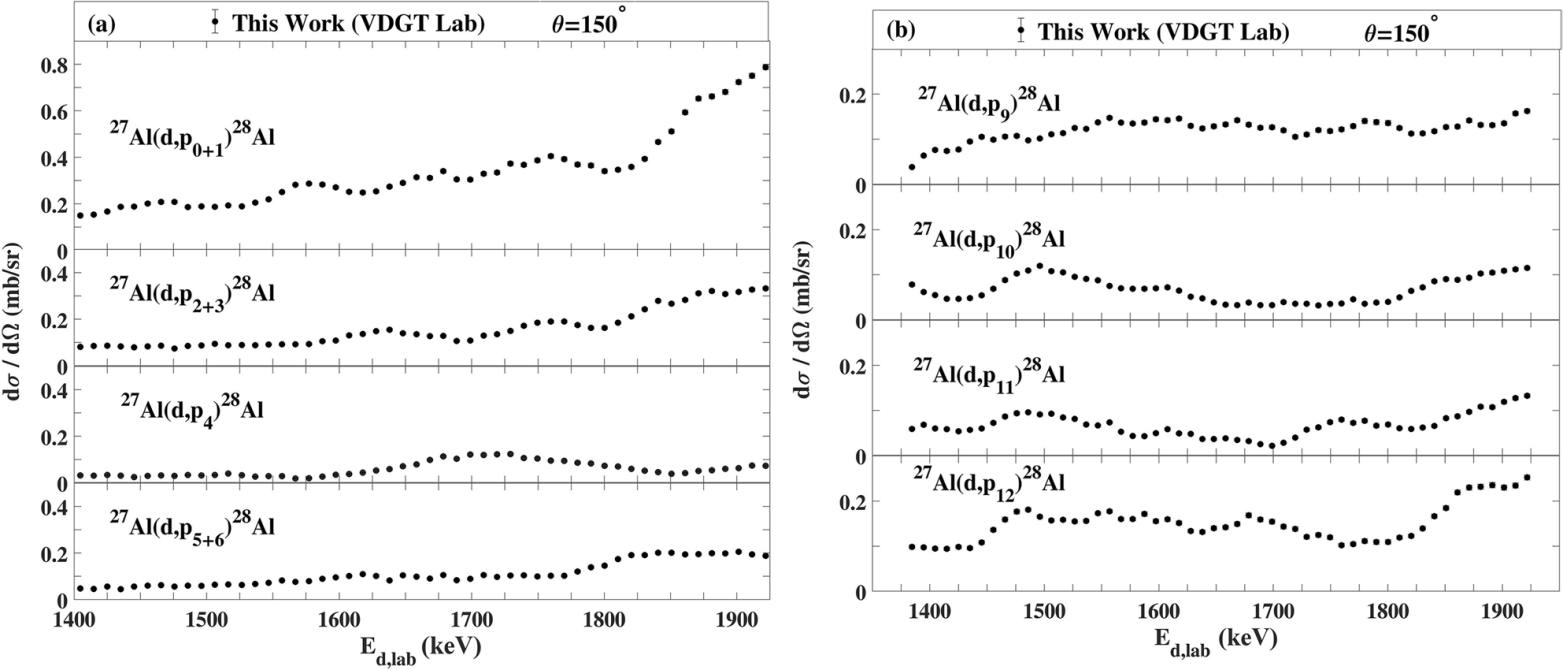
Differential cross section values for (**a**) ^27^Al(d,p_0-6_)^28^Al and (**b**) ^27^Al(d,p_9–12_)^28^Al reactions at scattering angle 150° at VDGT Lab in Tehran. The estimated uncertainties from counting statistics are smaller than the plotted symbols.

**Figure 6:**
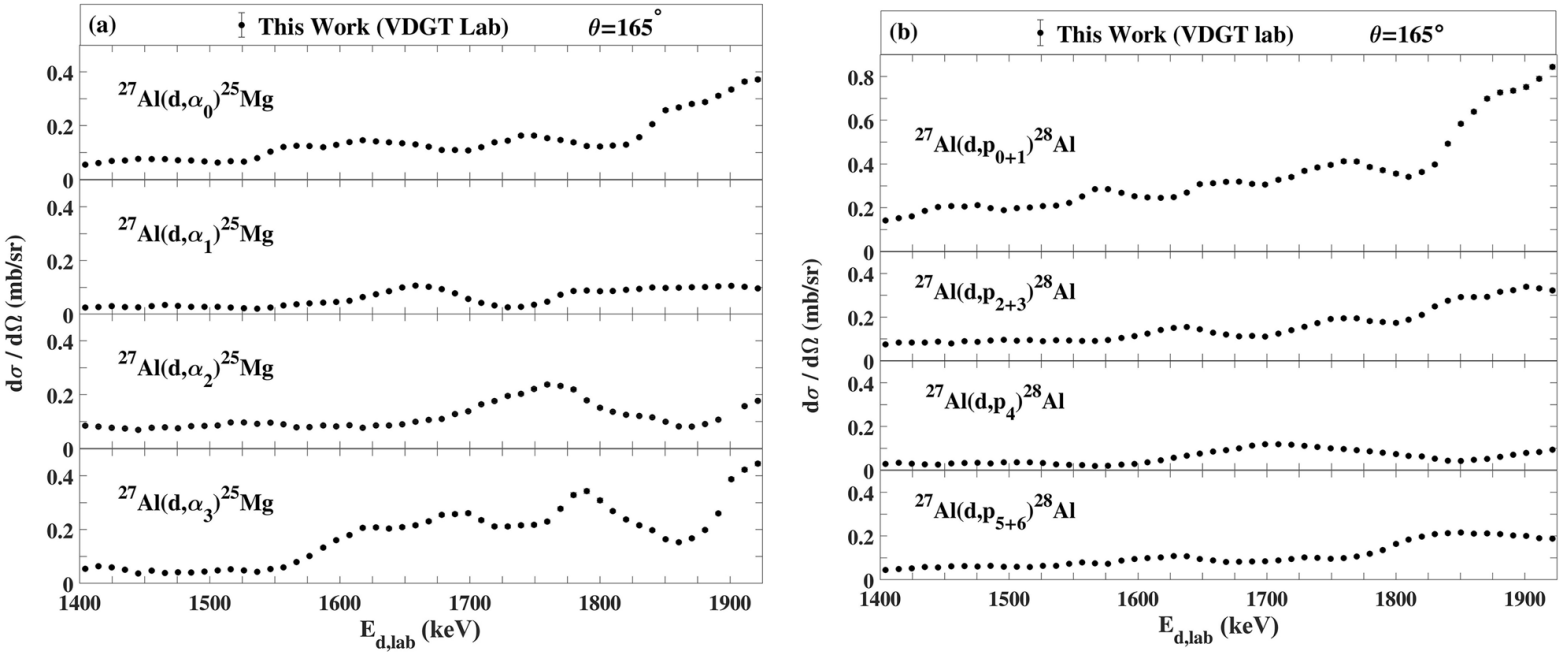
The differential cross sections at 165° measured at VDGT Lab in Tehran for the (**a**) ^27^Al(d,α)^25^Mg and (**b**) ^27^Al(d,p)^28^Al reactions. The estimated uncertainties from counting statistics are smaller than the plotted symbols.

**Figure 7:**
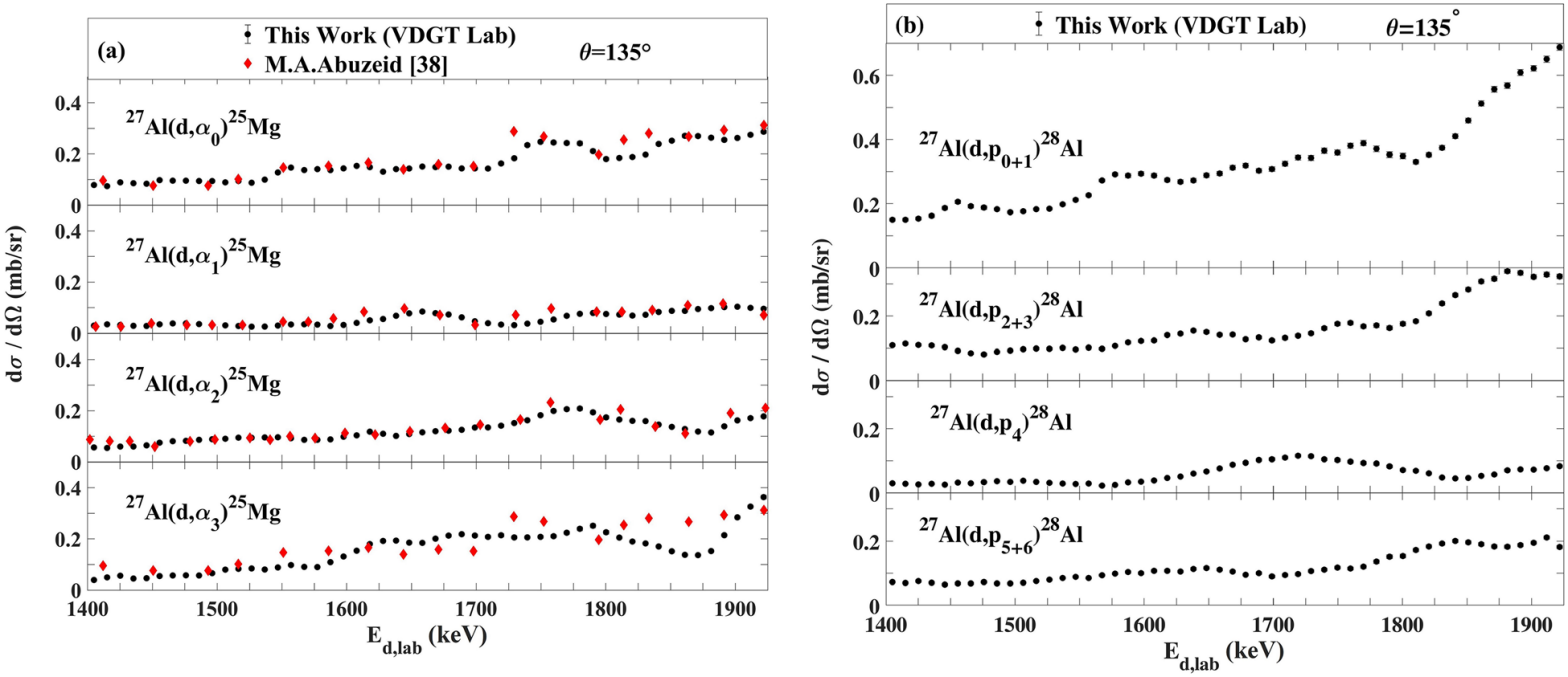
The differential cross sections measured at 135° at VDGT Lab in Tehran for the (**a**) ^27^Al(d,α)^25^Mg reactions together with the data from^38^, and (**b**) ^27^Al(d,p)^28^Al reactions. The estimated uncertainties from counting statistics are smaller than the plotted symbols.

The measured excitation functions at VDGT Lab at scattering angle 150° for the ^27^Al(d,α_0–3_)^25^Mg and ^27^Al(d,p_0–12_)^28^Al reactions are displayed in Figs. 4 and 5 and the excitation functions for the ^27^Al(d,α)^25^Mg and ^27^Al(d,p)^28^Al reactions at scattering angles 165° and 135° are shown in Figs. 6a,b, 7a,b. The only previous measurements of the ^27^Al(d,α) cross sections of which we are aware, apart from at 150° where there are several data sets that are discussed below, is that from^38^ for 135°. This data is indicated on Fig. 7a, however the values are rather sparse and show significant fluctuations. They were not further used in the present work.

### The differential cross section measurement at INSP lab in Paris

**Figure 8:**
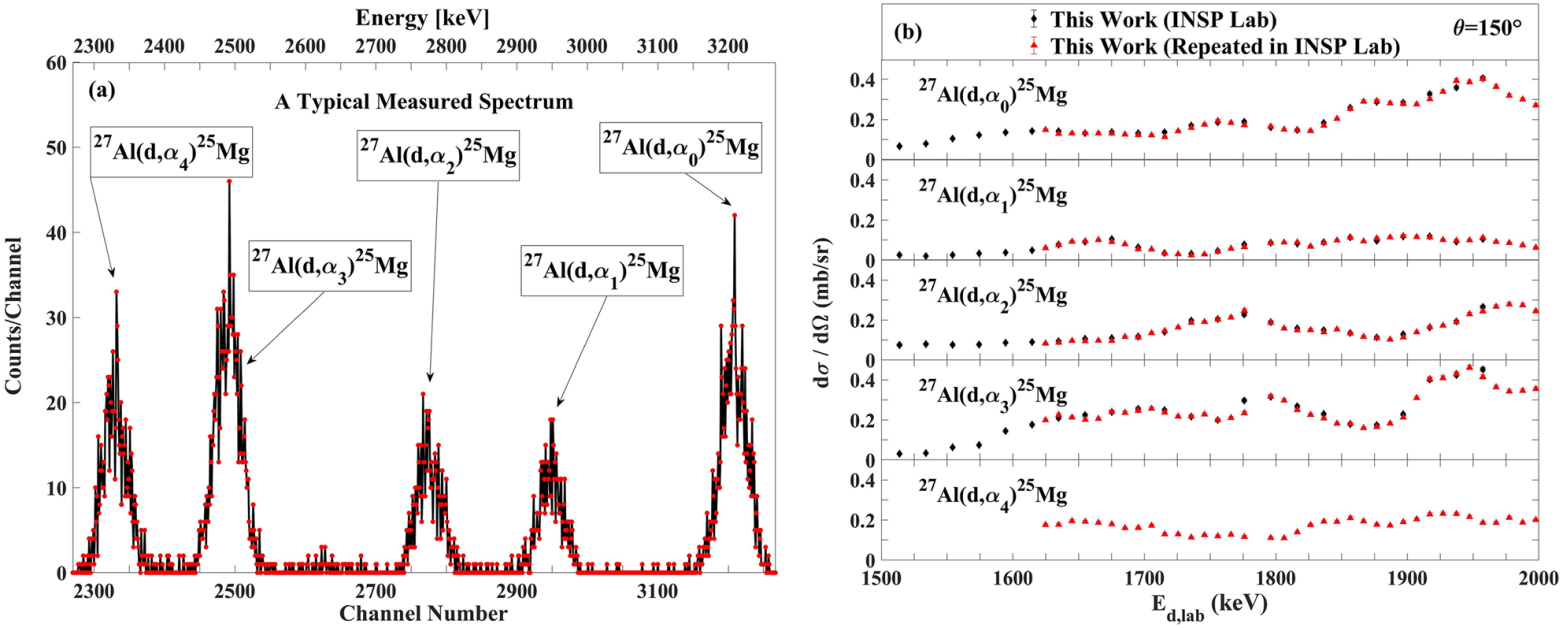
(**a**) A typical spectrum of a-particles for the ^27^Al(d,α)^25^Mg reaction, (**b**)The excitation functions for the ^27^Al(d,α)^25^Mg reaction at scattering angle 150° at INSP Lab in Paris. The estimated uncertainties from counting statistics are smaller than the plotted symbols.

**Figure 9:**
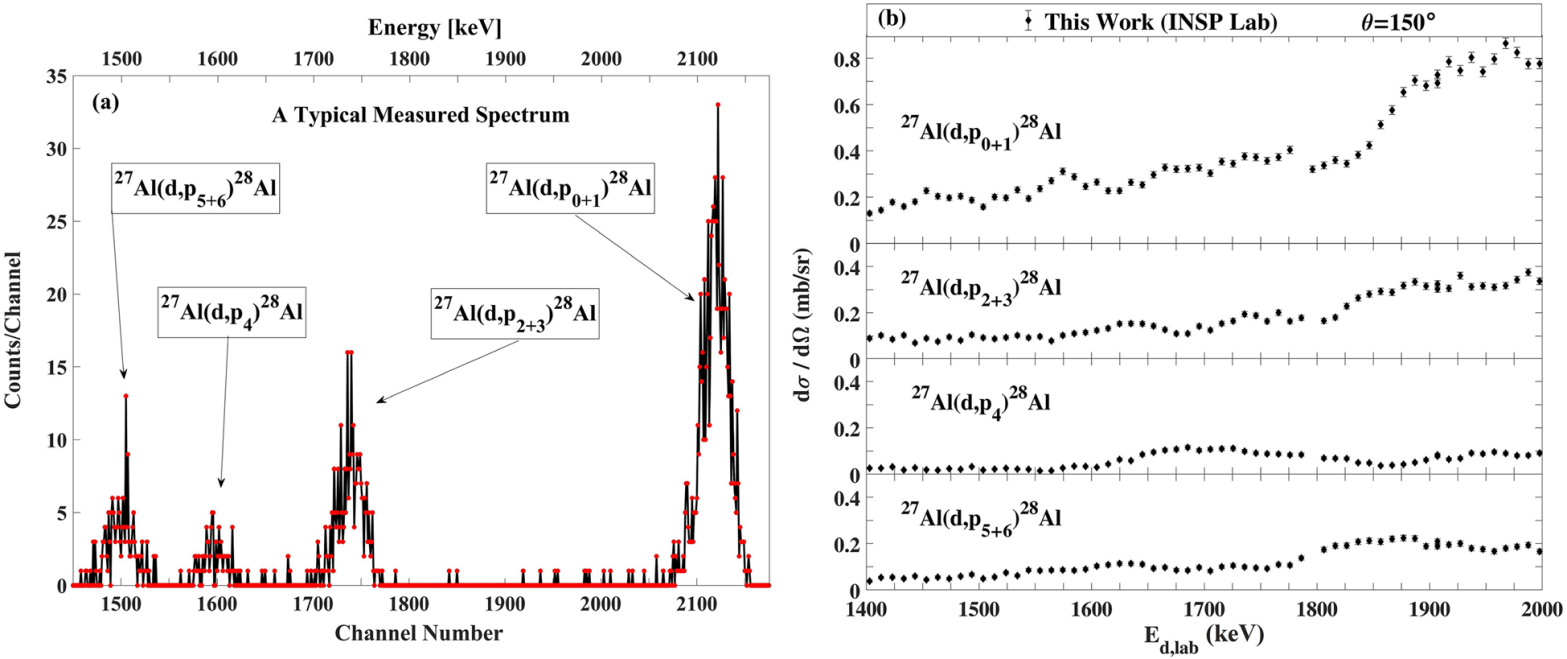
(**a**) A typical spectrum of protons for the ^27^Al(d,p)^28^Al reaction. (**b**) The excitation functions for the ^27^Al(d,p)^28^Al reaction at scattering angle 150° at INSP Lab in Paris. Where not visible, the estimated uncertainties from counting statistics are smaller than the plotted symbols.

The experiments were done with three different experimental setups, as follows: for (d,α) reaction measurements, we used the 4 µm-thick mylar in front of the 300 µm-thick surface barrier detector and repeated these measurements without mylar in front of the detector. For (d,p) reaction measurements, in the third configuration, a 100 µm-thick mylar was chosen in front of a 500 µm-thick pin diode detector at scattering angle of 150°. Typical experimental alpha particle and proton spectra from the ^27^Al(d,α)^25^Mg and ^27^Al(d,p)^28^Al reactions are shown in Figs. 8a and 9a, respectively.

The differential cross-sections were determined by using equation3, as following:

3 $$\left( {\frac{d\sigma }{{d\Omega }}} \right)_{{\theta ,E_{d} (Al)}} = \left( {\frac{d\sigma }{{d\Omega }}} \right)_{{\left. {\theta ,EBS,E_{d} (Au} \right)}}^{Au} \times \frac{{Y_{Al} }}{{Y_{Au} }} \times \frac{{N_{Au} }}{{N_{Al} }}$$

where *θ*, *Y*_*Al* and Au_, *E*_*d*_(*Au*), *E*_*d*_(*Al*) correspond to the scattering angle, integrated yields (*Au* and *Al*) as obtained from the﻿ experimental spectra, the energy at the surface of the Au layer, and the energy at half of the Aluminium thickness (including energy loss in the *Au* film), respectively. $\frac{{N_{Au} }}{{N_{Al} }}$ represents the areal density of *Au* versus *Al* present in the target, measured by RBS and calculated by equation[1], just replacing Ag by Au.

### Uncertainties

Using the measured $\frac{{N_{Au} }}{{N_{Al} }}$ ratio and normalizing the cross section measurement to the corresponding Rutherford cross-section, the uncertainties owing to solid angle, detector angular settings, dead time, and charge measurement were eliminated^39^. The uncertainty of the $\frac{{N_{Au} }}{{N_{Al} }}$ ratio was estimated to be about 3% according to the counting statistics. The corresponding statistical uncertainties of $\left( {\frac{{{\text{d}}\sigma }}{{{\text{d}}\Omega }}} \right)_{{\theta ,E_{d} ({\text{Al}})}}$ were estimated to be 4%-9% according to the error propagation formulas.

The measured differential cross section at INSP Lab with error bars at scattering angle 150° for the ^27^Al(d,α_0–3_)^25^Mg and ^27^Al(d,p_0–6_)^28^Al reactions are indicated in Figs. 8b and 9b.

## Discussion

### Correspondence between VDGT’s data and INSP’s data

**Figure 10:**
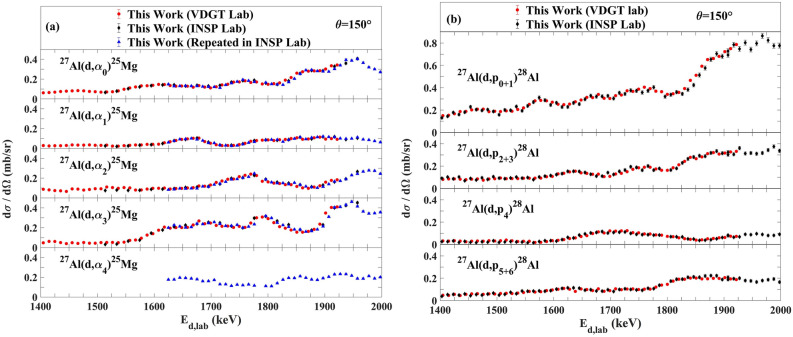
The comparison between our data from VDGT and from INSP at 150° for the (**a**) ^27^Al(d,α_0,1,2,3,4_)^25^Mg and (**b**) ^27^Al(d,p_0+1,2+3,4,5+6_)^28^Al reactions.

Figure 10 shows the comparison between our data from VDGT and from INSP. The results show that the data acquired at INSP Lab in Paris for ^27^Al(d,α_0,1,2,3,4_)^25^Mg and ^27^Al(d,p_0+1,2+3,4,5+6_)^28^Al are in acceptable agreement with data obtained at VDGT Lab at 150° scattering angle. Since these datasets were obtained under completely independent and somewhat different setups, this agreement is a strong indicator of the validity of the datasets. We further note that the measured target thicknesses correspond to deuteron energy losses of 8–10 keV in the Paris (INSP lab) samples and of 26–30 keV in the Teheran (VDGT lab) samples. This means that the measured cross sections are smoothed over these energy ranges. The very good correspondence between the two measurement sets also confirms that the cross sections are varying only slowly over these energy ranges.

### Comparison with previous studies

**Figure 11:**
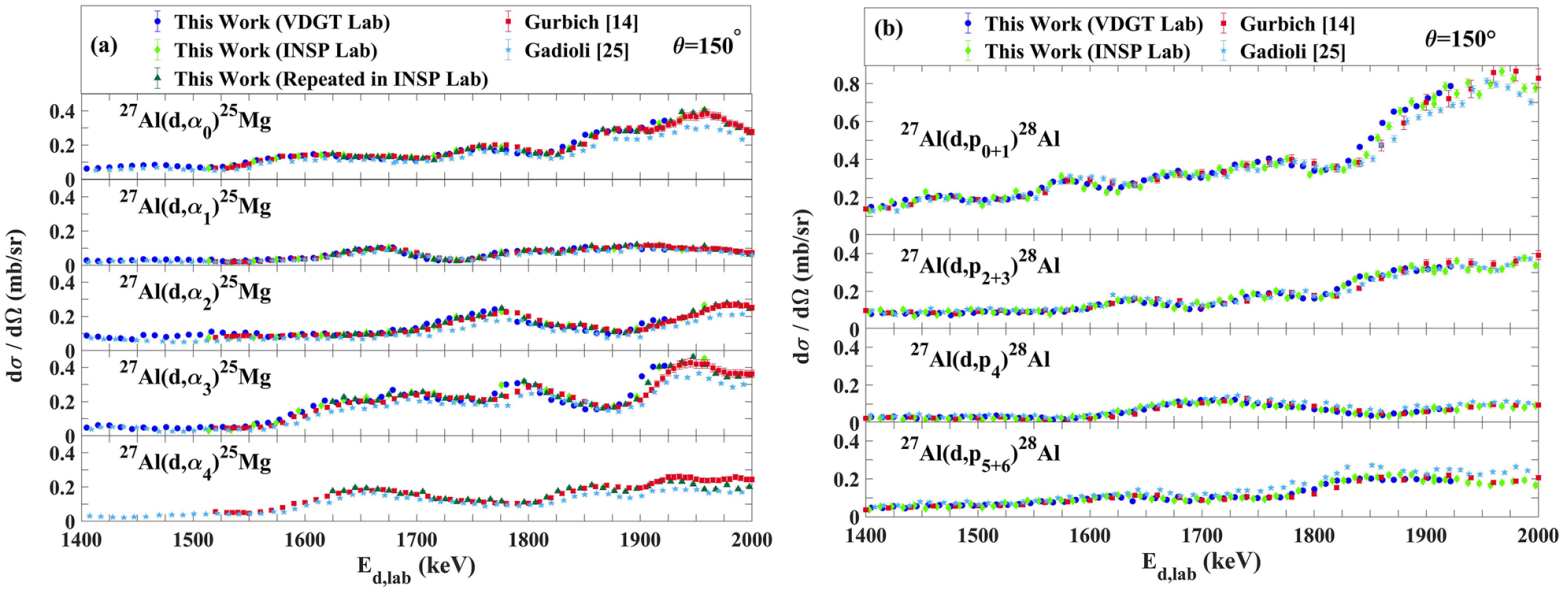
The comparison of our work with other data at 150° for (**a**) ^27^Al(d,α_0,1,2,3,4_)^25^Mg and (**b**) ^27^Al(d,p_0+1,2+3,4,5+6_)^28^Al reactions^14,25^.

**Figure 12:**
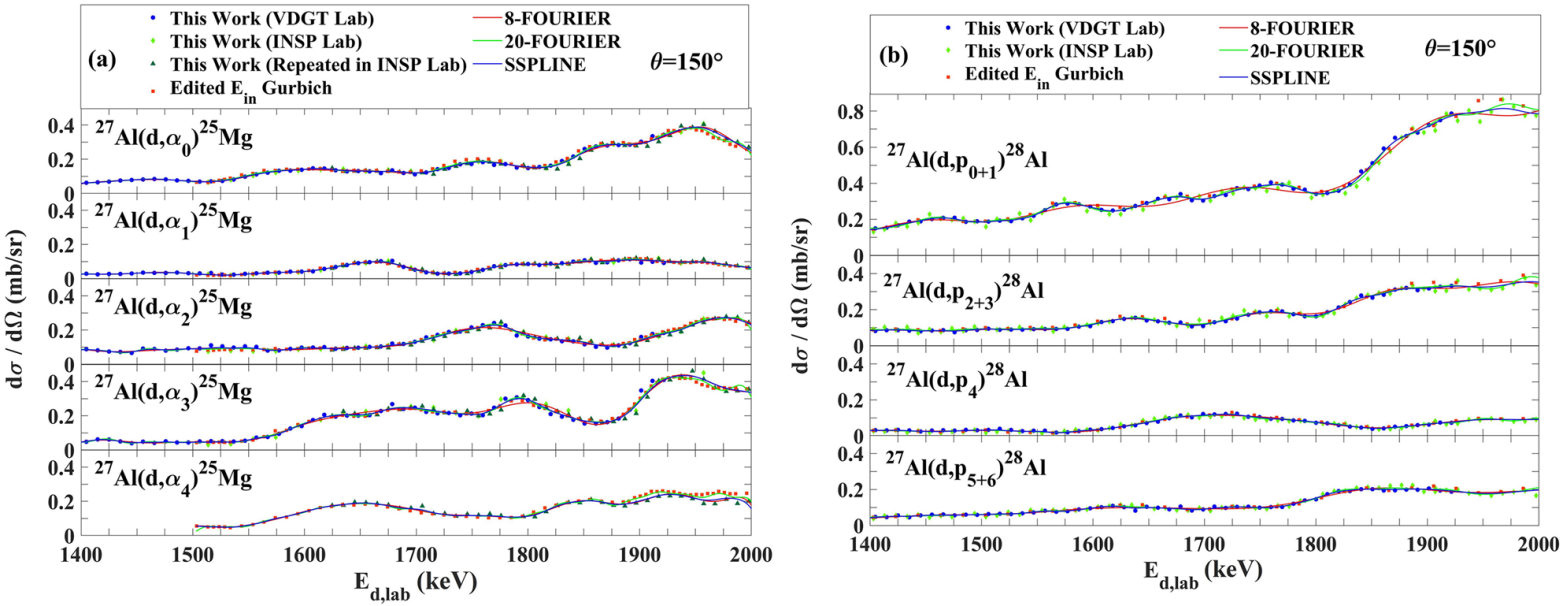
The comparison of different fitting methods for cross sections at 150° for (**a**) ^27^Al(d, α_0,1,2,3,4_)^25^Mg and (**b**) ^27^Al(d,p_0+1,2+3,4,5+6_)^28^Al reactions.

Although we are not aware of previous measurements for 135° (other than^38^, for (d,α), discussed above) and 165° scattering angles, several data sets exist for 150°. Comparison of the cross sections measured here with available aluminum (d,p) and (d,α) cross-sections at 150° is presented in Fig. 11. As mentioned in^14^, the measurements of Ref.^17^ barely overlaps with previous studies, and the data from^20^ are underestimated by a factor of about 2, so they are no longer considered here. The differential cross section values in Ref.^25^ show systematic differences from the other data, beyond the uncertainties given in the paper, both for ^27^Al(d,p_0+1,2+3,4,5+6_)^28^Al and ^27^Al(d, α_0,1,2,3,4_)^25^Mg. Data in Ref.^14^ are in acceptable agreement with our data except for a small energy shift. We attribute this energy shift to the energy loss of the incident beam in the target, which was not taken into account in^14^. The four retained datasets have been plotted in Fig. 12 for the 150° scattering, with the data from Ref.^14^ corrected for the energy loss in the target, using the target thickness supplied in that publication.

### Evaluation of 150° cross section measurements

Since there is no suitable nuclear reaction model for these reactions, the question arises as to how to represent the best averaged values of the data to propose a single recommended cross section at any energy within the measured range. We have taken the approach of fitting a freely chosen mathematical function to all of the data, weighted inversely according to their reported uncertainties.

The four retained datasets were therefore fitted with 8-term and 20-term Fourier series, and a spline interpolation algorithm, shown in Fig. 12, by MATLAB programming language.

**Figure 13:**
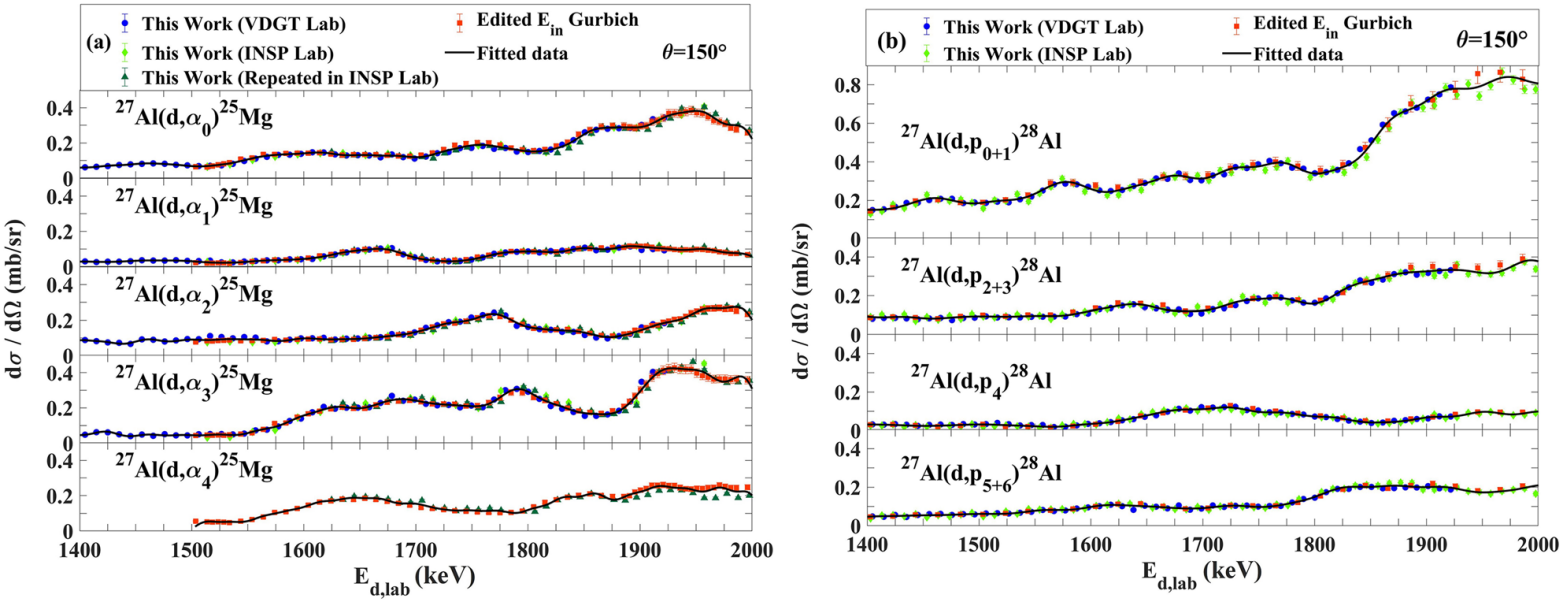
The fitted data for 150° for (**a**) ^27^Al(d,α_0,1,2,3,4_)^25^Mg and (**b**) ^27^Al(d,p_0+1,2+3,4,5+6_)^28^Al reactions.

The differences between the fitted functions are small but the 20-term Fourier series allows the data to be represented with good fidelity and not too many fitted parameters. This is shown in Fig. 13.

### Fourier series equations

One of the advantages of fitting the data with the Fourier series is that the theoretical function can be expressed easily and used as a reference model in future works. The actual Fourier series are written as follows^40^:

4 $$f\left( x \right) = a_{0} + \mathop \sum \limits_{{\left\{ {n = 1} \right\}}}^{N} a_{n} \cos \left( {n\omega x} \right) + b_{n} \sin \left( {n\omega x} \right)$$

Theoretically, the Fourier coefficients $a_{n}$ and $b_{n}$ can be evaluated as follows:

5 $$a_{0} = \frac{1}{T}\mathop \smallint \limits_{T} f\left( x \right)dx$$

6 $$a_{n} = \frac{2}{T}\mathop \smallint \limits_{T} f\left( x \right)\cos \left( {n\omega x} \right)dx$$

7 $$b_{n} = \frac{2}{T}\mathop \smallint \limits_{T} f\left( x \right)\sin \left( {n\omega x} \right)dx$$

Considering the N = 20 Fourier series, the coefficients for all of the cross sections reported here (in addition to the 150° collection) were calculated and are presented in Table 1. The goodness of fit is given by the coefficient of correlation R which describes the proportion of the variation of the data described by the fitted function^41^. R^2^ is above 0.96 for the highest cross sections, and above 0.9 even for the lower cross sections which are statistically noisier.

**Table 1:** The coefficients and Goodness of fitting for final fitting.

	^27^Al(d,α_0_)^25^Mg	^27^Al(d,α_1_)^25^Mg	^27^Al(d,α_2_)^25^Mg	^27^Al(d,α_3_)^25^Mg	^27^Al(d,α_4_)^25^Mg	^27^Al(d,p_0+1_)^25^Al	^27^Al(d,p_2+3_)^25^Al	^27^Al(d,p_4_)^25^Al	^27^Al(d,p_5+6_)^25^Al
a_0_	0.3102	0.1135	0.2474	0.3491	0.2877	0.7530	0.3259	0.1097	0.2070
a_1_	− 0.0212	− 0.0186	− 0.0270	− 0.0583	− 0.0268	0.1395	0.0736	0.0328	0.0591
a_2_	− 0.0601	− 0.0182	− 0.0077	− 0.0181	0.0684	− 0.1852	− 0.0587	− 0.0060	− 0.0334
a_3_	− 0.0442	− 0.0056	− 0.0422	− 0.0582	− 0.0113	0.0210	− 0.0086	0.0120	− 0.0027
a_4_	− 0.0247	− 0.0133	− 0.0319	− 0.0536	0.0087	0.0258	0.0107	0.0026	0.0033
a_5_	− 0.0155	0.0034	− 0.0113	0.0047	0.0016	− 0.0588	− 0.0198	− 0.0068	− 0.0178
a_6_	− 0.0039	− 0.0069	− 0.0059	0.0019	− 0.0090	0.0617	0.0117	0.0059	0.0036
a_7_	0.0126	0.0047	0.0033	− 0.0135	0.0116	− 0.0078	0.0052	− 0.0014	0.0004
a_8_	0.0001	− 0.0002	0.0015	0.0020	− 0.0061	− 0.0170	− 0.0006	− 0.0041	− 0.0056
a_9_	0.0019	0.0009	0.0069	0.0052	− 0.0021	0.0461	0.0059	0.0037	− 0.0009
a_10_	0.0043	0.0011	0.0050	0.0127	0.0027	− 0.0069	− 0.0074	− 0.0025	− 0.0033
a_11_	0.0069	− 0.0005	− 0.0013	0.0100	− 0.0010	− 0.0170	0.0007	− 0.0018	− 0.0031
a_12_	0.0021	− 0.0002	0.0020	0.0017	0.0033	0.0183	0.0082	0.0022	0.0039
a_13_	0.0000	0.0008	− 0.0023	0.0056	0.0000	− 0.0098	− 0.0018	− 0.0031	− 0.0014
a_14_	− 0.0010	− 0.0005	− 0.0015	− 0.0010	− 0.0022	− 0.0032	− 0.0011	0.0002	− 0.0023
a_15_	− 0.0038	− 0.0018	− 0.0029	− 0.0021	0.0038	0.0082	0.0038	0.0031	0.0020
a_16_	− 0.0049	− 0.0005	− 0.0041	− 0.0063	− 0.0016	− 0.0101	− 0.0004	− 0.0008	− 0.0003
a_17_	− 0.0042	− 0.0013	− 0.0036	− 0.0073	− 0.0007	0.0072	0.0024	0.0002	0.0011
a_18_	− 0.0040	− 0.0013	− 0.0039	− 0.0036	0.0026	0.0050	− 0.0016	0.0003	0.0010
a_19_	− 0.0024	− 0.0008	− 0.0022	− 0.0034	− 0.0030	0.0006	0.0005	0.0014	0.0005
a_20_	− 0.0002	0.0003	− 0.0013	− 0.0020	0.0022	0.0023	0.0009	0.0012	− 0.0005
b_1_	− 0.0945	− 0.0291	− 0.0502	− 0.1104	0.0425	0.2519	0.0933	0.0164	0.0501
b_2_	− 0.0442	− 0.0097	− 0.0099	− 0.0662	− 0.0309	0.0331	0.0389	− 0.0108	0.0241
b_3_	0.0088	0.0164	− 0.0263	− 0.0033	− 0.0160	− 0.1105	− 0.0390	− 0.0272	− 0.0113
b_4_	0.0310	− 0.0022	0.0238	0.0313	0.0179	0.0541	0.0216	− 0.0001	0.0219
b_5_	0.0098	0.0075	0.0161	0.0312	− 0.0160	− 0.0433	− 0.0028	− 0.0055	0.0041
b_6_	0.0208	0.0009	0.0211	0.0000	− 0.0014	− 0.0438	− 0.0139	0.0006	− 0.0099
b_7_	0.0073	0.0058	0.0126	0.0169	0.0120	0.0460	− 0.0006	0.0081	0.0049
b_8_	0.0029	0.0025	0.0082	0.0093	− 0.0113	− 0.0247	− 0.0137	− 0.0043	− 0.0023
b_9_	0.0058	0.0008	0.0083	0.0130	0.0047	− 0.0094	− 0.0005	0.0017	− 0.0015
b_10_	0.0037	− 0.0003	− 0.0022	0.0028	− 0.0020	0.0257	0.0002	0.0028	0.0020
b_11_	− 0.0031	− 0.0011	− 0.0033	− 0.0082	− 0.0015	− 0.0204	− 0.0086	− 0.0036	− 0.0032
b_12_	− 0.0043	0.0011	− 0.0029	− 0.0040	0.0029	− 0.0006	0.0016	0.0011	0.0000
b_13_	− 0.0055	− 0.0017	− 0.0059	− 0.0088	− 0.0048	0.0176	0.0050	0.0014	0.0021
b_14_	− 0.0052	− 0.0017	− 0.0049	− 0.0072	0.0013	− 0.0103	− 0.0049	− 0.0024	− 0.0021
b_15_	− 0.0041	− 0.0015	− 0.0053	− 0.0062	0.0023	0.0020	0.0013	0.0016	0.0001
b_16_	− 0.0022	− 0.0002	− 0.0007	− 0.0040	− 0.0041	0.0034	0.0003	0.0020	0.0018
b_17_	− 0.0008	− 0.0010	− 0.0005	0.0008	0.0045	− 0.0070	0.0002	− 0.0008	− 0.0015
b_18_	0.0014	0.0002	0.0017	0.0017	0.0002	0.0064	0.0033	0.0003	0.0002
b_19_	0.0018	0.0012	0.0022	0.0029	− 0.0026	− 0.0009	− 0.0012	− 0.0006	0.0010
b_20_	0.0022	0.0002	0.0004	0.0020	0.0033	0.0014	0.0021	0.0009	0.0007
*ω* = 2π/T									
*ω*	0.0092	0.0092	0.0092	0.0092	0.0113	0.0071	0.0071	0.0071	0.0071
Goodness of fitting									
R^2^	0.9767	0.9681	0.949	0.965	0.9139	0.9657	0.9699	0.947	0.9751

## Benchmarking the evaluated cross sections

The measured cross sections are validated by a benchmarking exercise^42,43^, in which the cross sections are used in an independent experiment. For NRA cross sections, this almost always consists in obtaining thick target NRA spectra from a well-defined (often mono-elemental) target and verifying the extent to which the measured cross sections reproduce the observed spectra when included in an established NRA simulator such as SIMNRA. To our knowledge, only the ^27^Al(d,p)^28^Al cross-sections have been benchmarked, by obtaining and fitting thick target spectra, for just one incident energy and one scattering angle^14^, whilst there have been no benchmarking experiments at all for ^27^Al(d,α)^25^Mg cross sections.

The goal of the present work is to benchmark the ^27^Al(d,p)^28^Al and ^27^Al(d,α)^25^Mg cross sections at three scattering angles, 135°, 150°, and 165°, and with various incident beam energies in two different laboratories.

For the experimental part of benchmarking, charged particle spectra were obtained from a thick pure aluminium target under deuteron irradiation. For the simulation part of benchmarking, the SIMNRA 7.03 code is applied with the Chu and Yang straggling model and Ziegler/Biersack stopping power.

### Benchmarking of measured data for ^27^Al(d,p)^28^Al and ^27^Al(d,α)^***25***^Mg reactions at VDGT lab in Tehran

**Figure 14:**
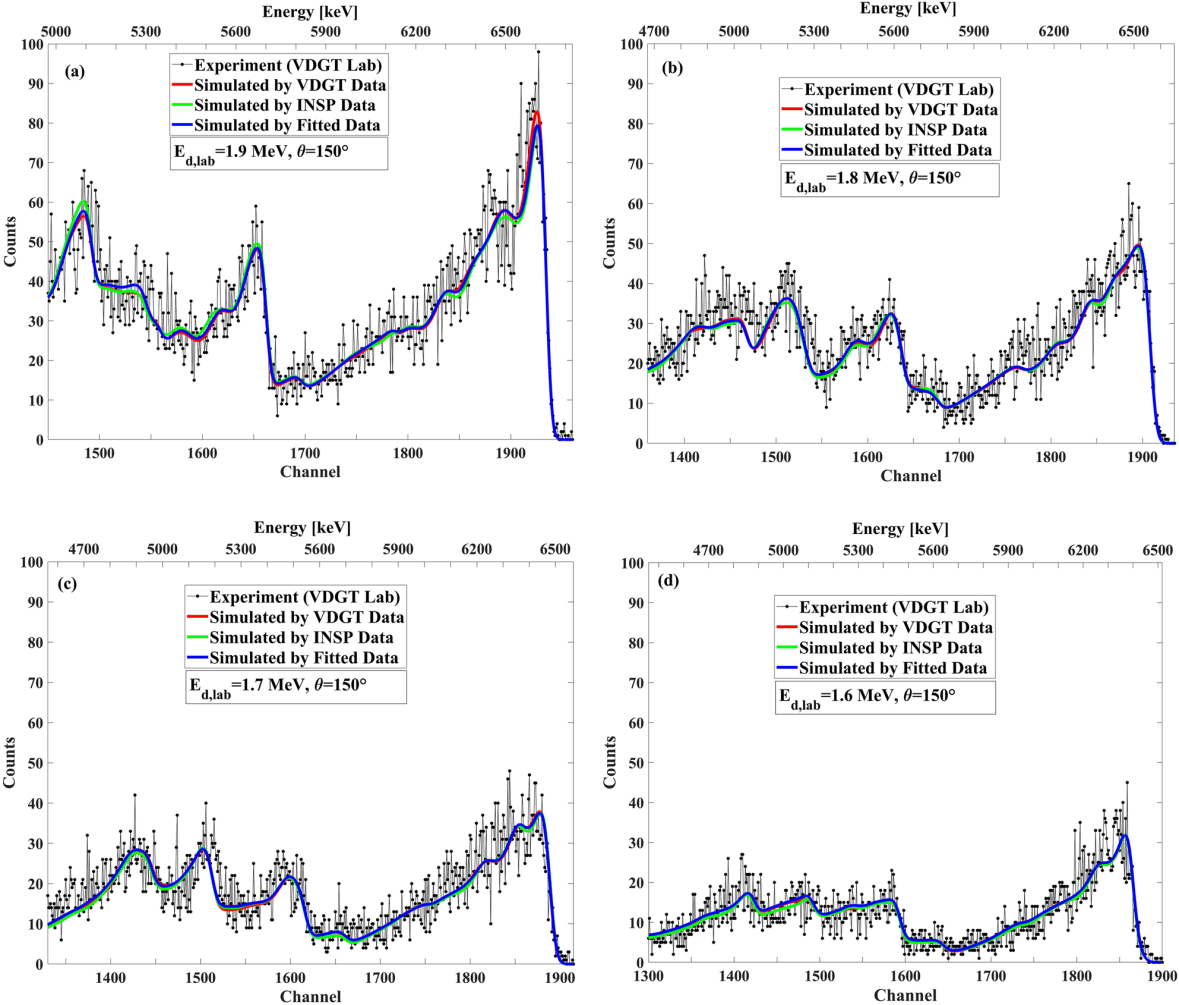
The benchmarking result at VDGT Lab in Tehran with different data sets at 150° and different incident deuteron energies, (**a**) 1.9 MeV, (**b**) 1.8 MeV, (**c**) 1.7 MeV and (**d**) 1.6 MeV.

**Figure 15:**
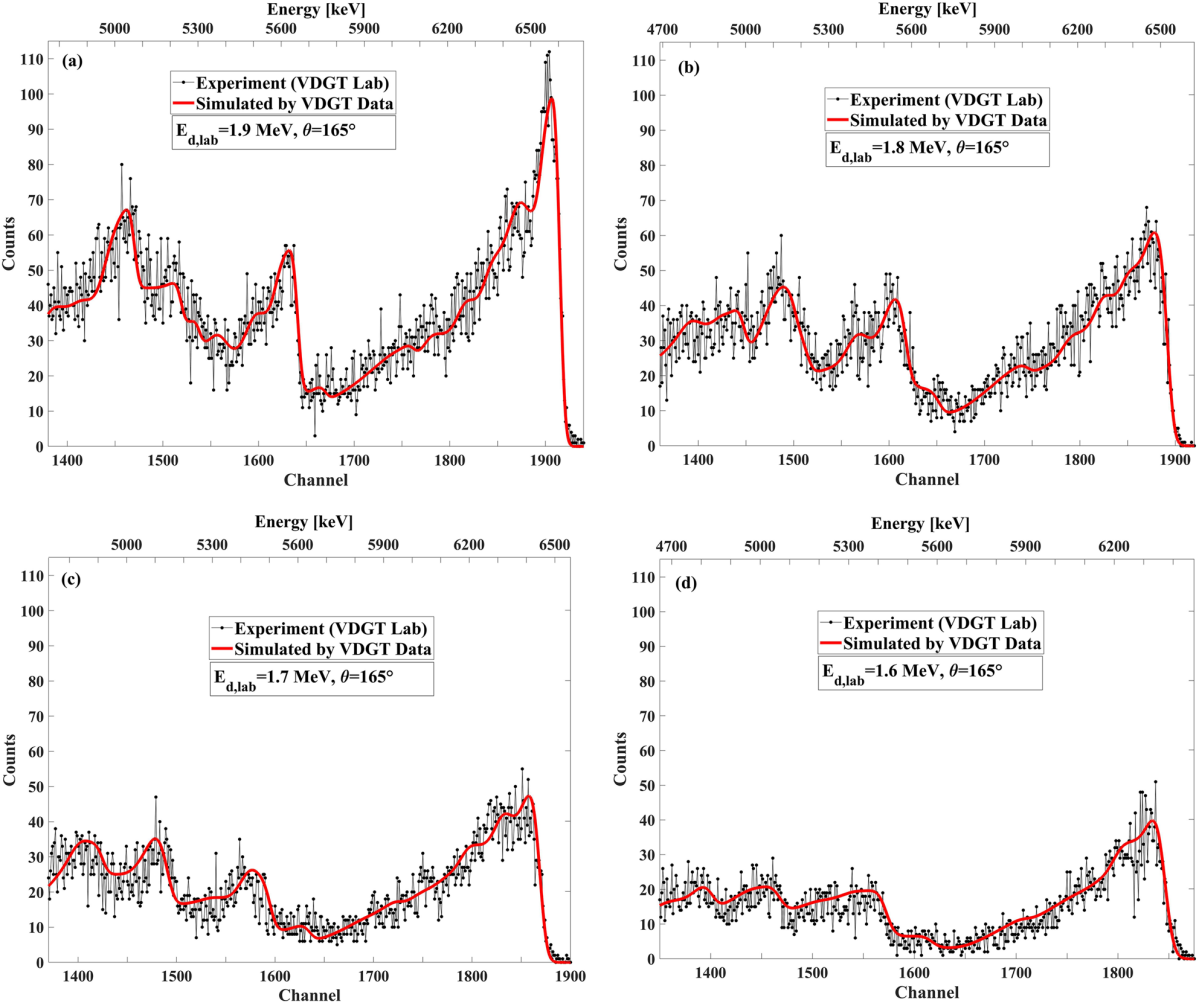
The benchmarking result at VDGT Lab in Tehran with measured VDGT data at 165° and different incident deuteron energies, (**a**) 1.9 MeV, (**b**) 1.8 MeV, (**c**) 1.7 MeV and (**d**) 1.6 MeV.

**Figure 16:**
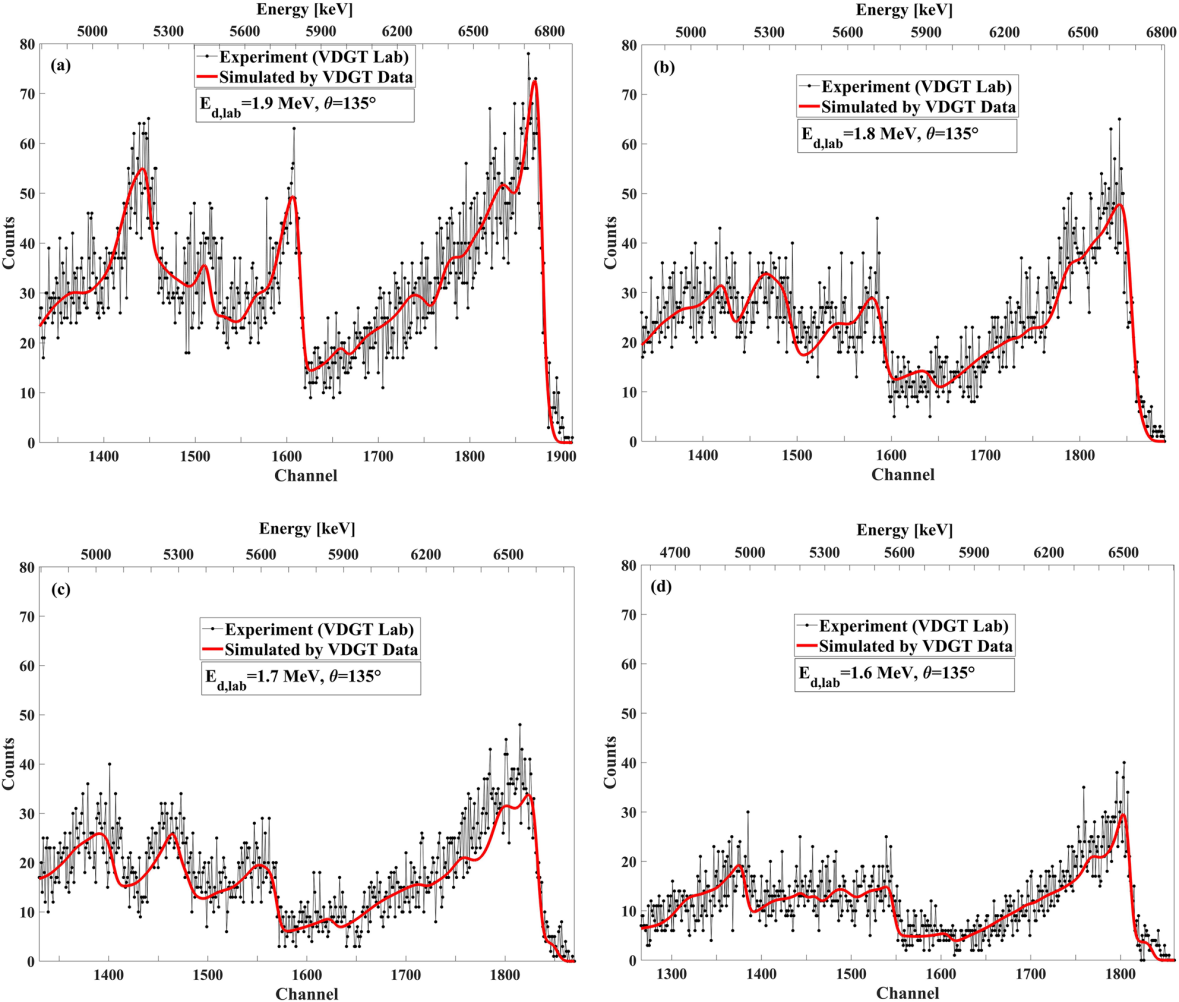
The benchmarking result at VDGT Lab in Tehran with measured VDGT data at 135° with different incident deuteron energies, (**a**) 1.9 MeV, (**b**) 1.8 MeV, (**c**) 1.7 MeV and (**d**) 1.6 MeV.

For benchmarking the ^27^Al(d,p)^28^Al and ^27^Al(d,α)^25^Mg cross sections at VDGT Lab in Tehran, we used a thick pure Al target with a thin Ag layer deposited on it for the self-normalization process. No mylar was used in front of the detector. Other experimental parameters are as explained in “Experimental setup and procedure at VDGT” section. The results of benchmarking for E_d_ = 1600–1900 keV at the scattering angles of 135°, 150° and165°, with an incident energy interval of 100 keV, are shown in Figs. 14, 15, and 16, respectively. At 150°, the three cross section data sets (VDGT data, INSP data, and fitted data) have been incorporated into the SIMNRA library. Overall, the agreement between the simulated and measured spectra is satisfying.

### Benchmarking of measured data for ^27^Al(d,p)^28^Al reactions at INSP lab in Paris

**Figure 17:**
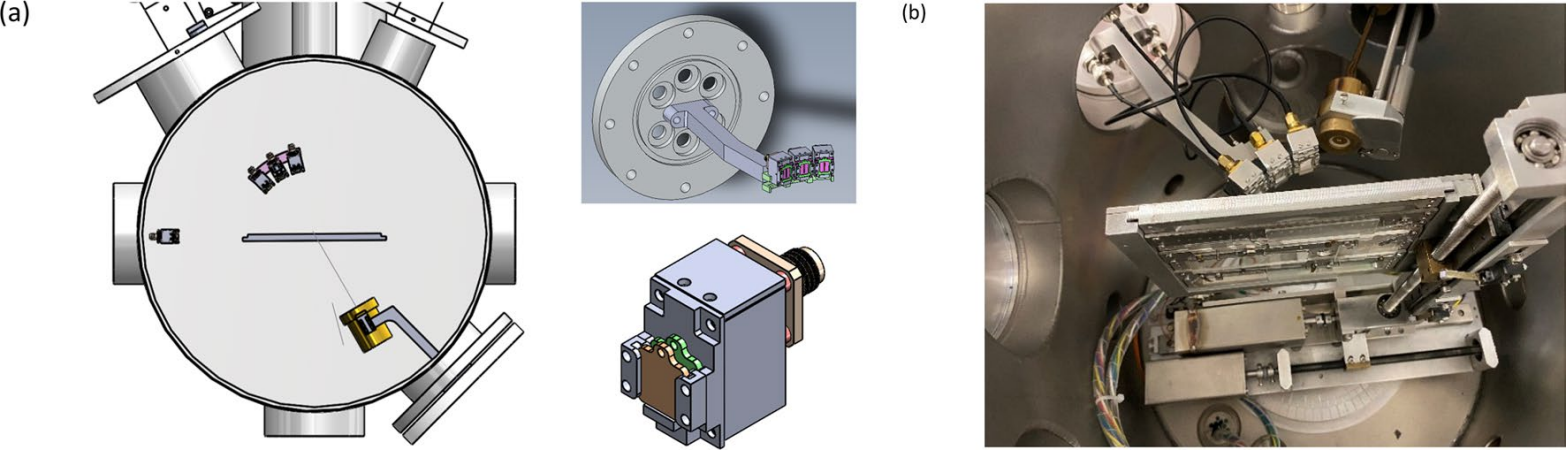
(**a**) Simulation of multi-detector holder kindly provided by S. Steydli, INSP, (**b**) set up of three pin detectors with 100 µm mylar in front of the detector.

For benchmarking the ^27^Al(d,p)^28^Al cross sections at INSP Lab in Paris, Au rather than Ag deposited on the thick pure Aluminum target acted as the internal reference. Moreover, a new multi-holder detector at INSP lab was designed (Fig. 17), which included three 1 cm^2^ Hammamatsu S3590-09 PIN diodes, with depletion depth of 300 μm, as detectors at 135°, 150° and 165°. A 100 µm mylar foil in front of each detector ensured the elimination of the alpha particles from the spectrum.

**Figure 18:**
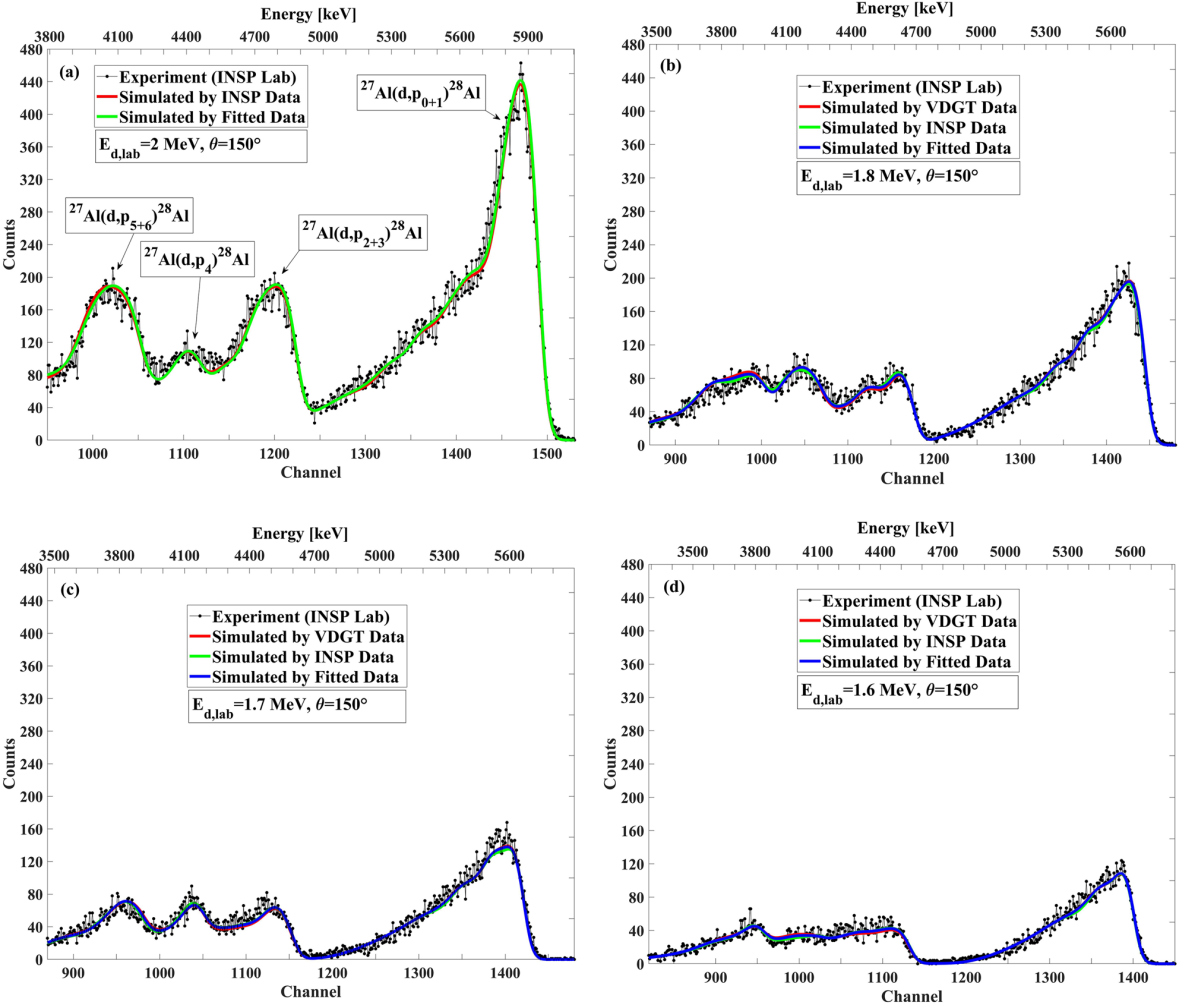
The benchmarking result at INSP Lab in Paris with different data set at 150° for incident deuteron energies of (**a**) 2 MeV, (**b**) 1.8 MeV, (**c**) 1.7 MeV, (**d**) 1.6 MeV.

**Figure 19:**
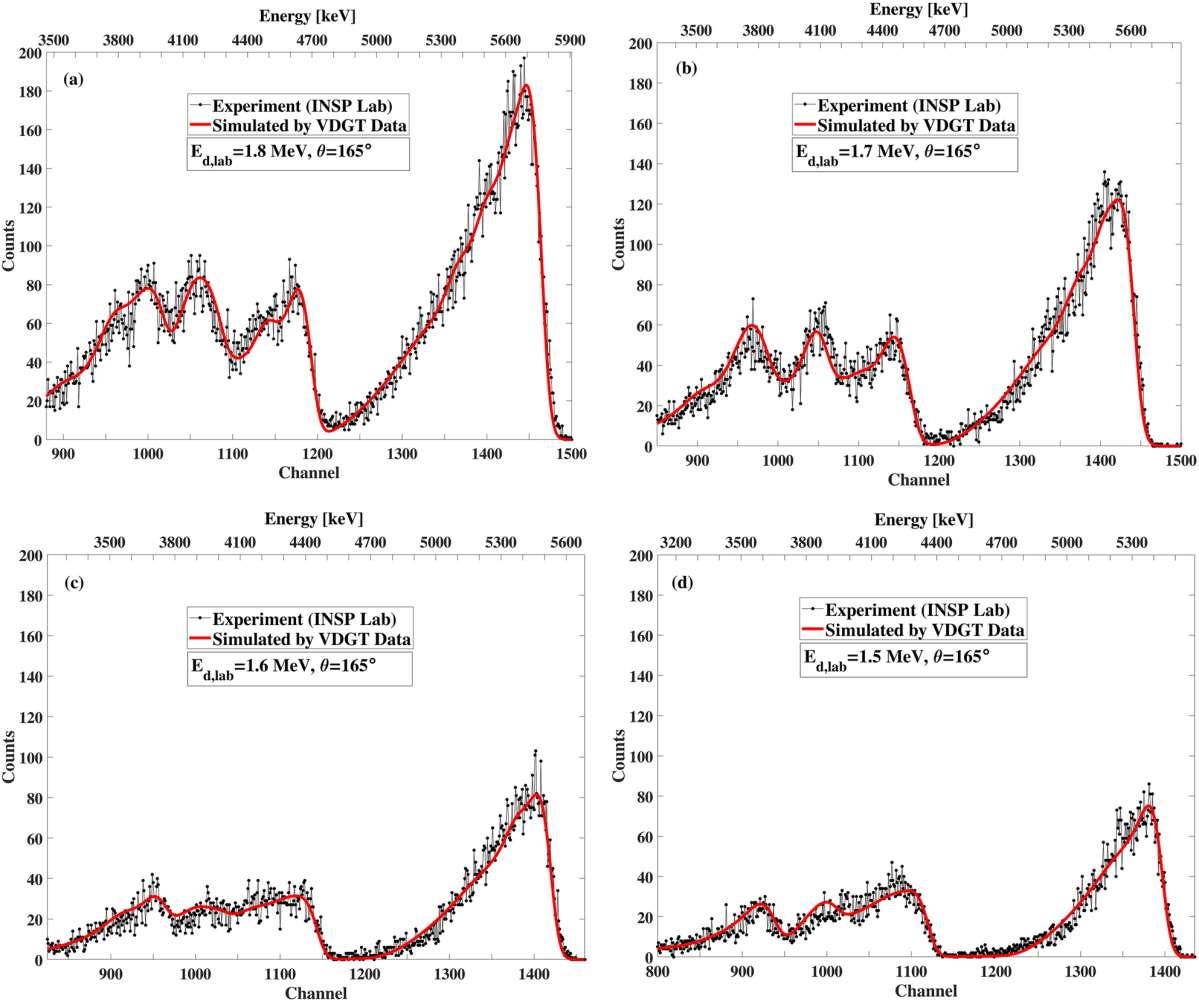
The benchmarking result at INSP Lab in Paris with measured VDGT data at 165° for different incident deuteron energies of (**a**) 1.8 MeV, (**b**) 1.7 MeV, (**c**) 1.6 MeV, (**d**) 1.5 MeV.

**Figure 20:**
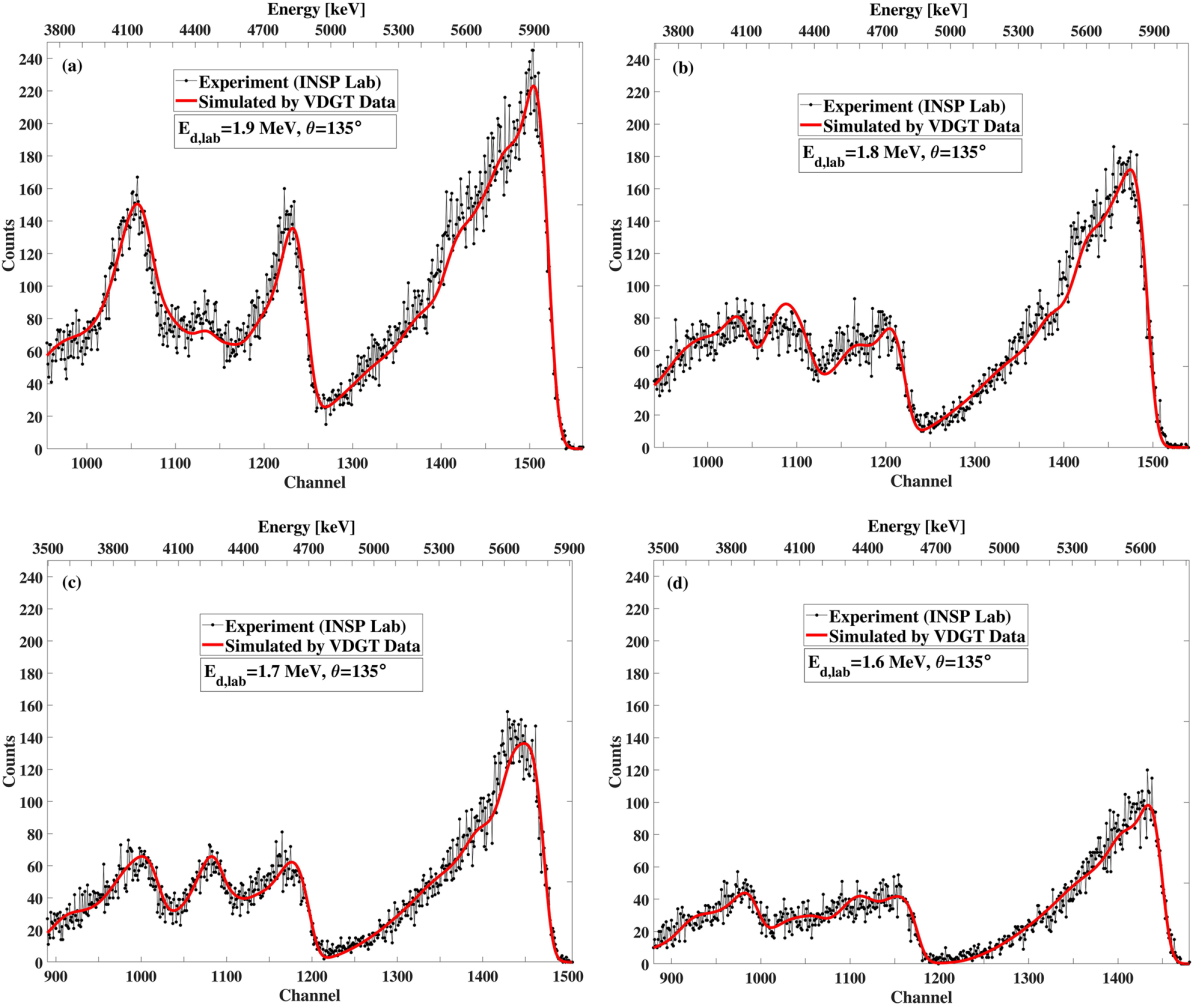
The benchmarking result at INSP Lab in Paris with measured VDGT data at 135° for different incident deuteron energies of (**a**) 1.9 MeV, (**b**) 1.8 MeV, (**c**) 1.7 MeV, (**d**) 1.6 MeV.

The benchmarking results are displayed in Fig. 18 for E_d_ = 1700–2000 keV with an energy step of 100 keV, at the scattering angle of 150° for VDGT data, INSP data, and fitted data sets. Figures 19 and 20 present the benchmarking results for E_d_ = 1500–1800 keV with an energy step of 100 keV at the scattering angles of 165° and 135°, respectively. The simulation at these angles employed the measured VDGT cross section data. In general, the correspondence between the simulations and the measured thick target spectra was very satisfying.

The range of measured cross-sections used for simulating the spectra was 1.4–2 MeV. After energy loss in the Al target, whenever E_d,Lab_ falls below the minimum energy of the measured cross section (1.4 MeV), the corresponding simulation of measured cross-sections has no value and SIMNRA considers its value equal to zero. In this case, for the ^27^Al(d,p_0+1_) reaction we manually inserted widely spaced cross section values for energies below 1.4 MeV so that the measured thick target spectra in the energy range from 4700 to 5000 keV were reproduced by the simulations. Similar inclusions were made for the ^27^Al(d,p_2+3_, p_4_, p_5+6_) cross sections in the relevant energy ranges. These cross section values are certainly of the right amplitude, but because of the energy straggling of the incident beam at these depths in the target, in this energy range the proposed cross section would not faithfully represent any fine structure. In order to differentiate between the cross sections measured by the thin targets and incarnated by the N = 20 Fourier series, from the values inferred from the benchmarking experiments, the two data sets are loaded separately into IBANDL.

## Conclusions

The ^27^Al(d,p_0+1,2+3,4,5+6_)^28^Al and ^27^Al(d, α_0,1,2,3,4_)^25^Mg reaction cross sections were measured at VGDT (Tehran) on thin self-supporting aluminium targets for incident deuteron energies in the range 1.4–2 MeV at 135°, 150°, and 165° laboratory scattering angles. The measurements for 150° were independently repeated on the SAFIR platform at INSP and showed close agreement with the VDGT data. The cross sections at 150° were evaluated with existing data sets and an N = 20 Fourier series fit is proposed to embody the evaluated cross section. The evaluated cross-sections have been benchmarked through a series of thick target spectra of charged particles induced by deuteron beams from a pure aluminium target, under various detection conditions at both VDGT and INSP. The overall agreement between the spectra simulated by SIMNRA and the measured benchmarking spectra is most satisfying and validates the evaluated cross sections presented here. We therefore recommend the use of these evaluated cross sections for use in NRA. We note that the recommended cross sections for 135° and 165° are the first ^27^Al(d,p) and ^27^Al(d,α) cross sections to be benchmarked at these angles for NRA.
